# Accelerated theta-burst repetitive transcranial magnetic stimulation for depression in South Africa: A series of nine cases

**DOI:** 10.4102/sajpsychiatry.v24i0.1272

**Published:** 2018-10-01

**Authors:** Erine Bröcker, Leigh van den Heuvel, Soraya Seedat

**Affiliations:** 1 Faculty of Health Sciences, Stellenbosch University, South Africa

## Abstract

**Introduction:**

This case series documents local experience using accelerated theta-burst repetitive transcranial magnetic stimulation (rTMS) as a supplementary treatment for depression in both major depressive disorder (MDD) and bipolar disorder (BD).

**Methods:**

Nine consenting patients (MDD = 7; BD [major depressive episode] = 2) received the accelerated theta-burst protocol consisting of three magnetic pulses delivered 20 ms apart and repeatedly delivered every 200 ms, resulting in a 5 Hz theta rhythm over the left dorsolateral prefrontal cortex (DLPFC). Treatment comprised 20 sessions delivered over 8 days. Accelerated theta burst rTMS treatment provides more stimuli over a shorter period of time, thus potentially increasing feasibility and cost-effectiveness. Improvement was monitored using the Centre for Epidemiological Studies Depression (CES-D) scale and the Clinical Global Impression (CGI) scale at baseline, day 5 and day 8 of rTMS treatment. All patients remained on their prescribed medication for the duration of rTMS treatment. We performed a Wilcoxon matched-pairs signed rank test to determine whether there was a difference in scores from baseline to post-treatment. The CES-D scores decreased significantly from baseline (Mdn 38.0; IQR 31.0; 51.0) to post-treatment (Mdn 17.0; IQR 11.0; 28.5; *Z* = -2.55, *p* = 0.011). The CGI severity scores also decreased significantly between baseline (Mdn 4.0; IQR 4.0; 5.0) and post-treatment (Mdn 3.0; IQR 3.0; 4.0; *Z* = -2.43, *p* = 0.015).

**Results:**

Five patients demonstrated at least a 50% symptom reduction on the CES-D scale. The most commonly reported adverse effect was mild headache, which lasted a few hours during and post-rTMS treatment.

**Conclusion:**

A limitation of these findings is that this was a small case series without a control arm; however, the findings suggest that the accelerated theta burst rTMS protocol for depression was well tolerated with most patients also experiencing symptomatic improvement by day 8.

